# A high sensitivity ZENK monoclonal antibody to map neuronal activity in *Aves*

**DOI:** 10.1038/s41598-020-57757-6

**Published:** 2020-01-22

**Authors:** Gregory Charles Nordmann, Erich Pascal Malkemper, Lukas Landler, Lyubov Ushakova, Simon Nimpf, Robert Heinen, Stefan Schuechner, Egon Ogris, David Anthony Keays

**Affiliations:** 1 Research Institute of Molecular Pathology, Vienna Biocenter (VBC), Campus-Vienna-Biocenter 1, 1030 Vienna, Austria; 2 Monoclonal Antibody Facility, Max Perutz Labs, Medical University of Vienna, Dr. Bohr-Gasse 9, 1030 Vienna, Austria

**Keywords:** Immunohistochemistry, Neuroscience

## Abstract

The transcription factor ZENK is an immediate early gene that has been employed as a surrogate marker to map neuronal activity in the brain. It has been used in a wide variety of species, however, commercially available antibodies have limited immunoreactivity in birds. To address this issue we generated a new mouse monoclonal antibody, 7B7-A3, raised against ZENK from the rock pigeon (*Columba livia*). We show that 7B7-A3 labels *cl*ZENK in both immunoblots and histological stainings with high sensitivity and selectivity for its target. Using a sound stimulation paradigm we demonstrate that 7B7-A3 can detect activity-dependent ZENK expression at key stations of the central auditory pathway of the pigeon. Finally, we compare staining efficiency across three avian species and confirm that 7B7-A3 is compatible with immunohistochemical detection of ZENK in the rock pigeon, zebra finch, and domestic chicken. Taken together, 7B7-A3 represents a useful tool for the avian neuroscience community to map functional activity in the brain.

## Introduction

Over the past century *Aves* have served as powerful models to study vertebrate cognitive and sensory neurobiology^1^. It has been shown that crows and cockatoos can develop tools to solve complex tasks^2–4^, pigeons can memorize abstract visual patterns^5,6^, songbirds are able to learn conspecific and novel vocalizations^7,8^, and robins rely on magnetic fields for long-range navigation^9,10^. It is apparent from these studies that the avian brain processes information from a myriad of sensory cues allowing it to drive an array of complex behaviours.

The quantitation of immediate early gene (IEG) expression is a well established methodology to map the neuronal networks that integrate this information. IEGs represent a class of genes characterized by a rapid rise in expression levels upon sustained cell stimulation and are therefore linked to synaptic activity in the brain^11,12^. One commonly used IEG is the transcription factor ZENK (an acronym for *zif-268*, *egr-1*, *ngfi-a*, *krox-24*)^13–16^. Upon expression, this zinc finger protein regulates the transcription of downstream targets, thereby modulating synaptic long-term adaptations to incoming information^17^. ZENK mRNA is induced rapidly in response to stimulation^18^, followed by peak protein expression approximately 1–2 hours after stimulus onset^19,20^. It should be noted, however, that not all neuronal populations in the bird brain express ZENK^19,21–23^, and that ZENK induction is dependent on stimulus context^24,25^ or neuromodulatory factors such as the activity of the noradrenergic system^26^.

To date avian studies relying on ZENK expression have employed *in situ* hybridization, or a commercial polyclonal antibody raised against the rabbit ZENK homologue EGR-1 (C-19)^19,27–30^. While C-19 has been widely used, it displays high batch-to-batch variation, requires laborious amplification methods in some species^27,28^, and its production has now been discontinued by the provider. In order to establish a reliable alternative to C-19 we set out to generate a ZENK antibody specifically for *Aves*. Here, we describe the generation and validation of a new ZENK monoclonal antibody, 7B7-A3, raised in mice against a ZENK peptide from the rock pigeon (*Columba livia*). We show that 7B7-A3 labels ZENK in pigeons, zebra finches, and chickens, and it serves as a useful marker to detect auditory-induced neuronal activation.

## Results

### ZENK antibody generation and validation

**Figure 1:**
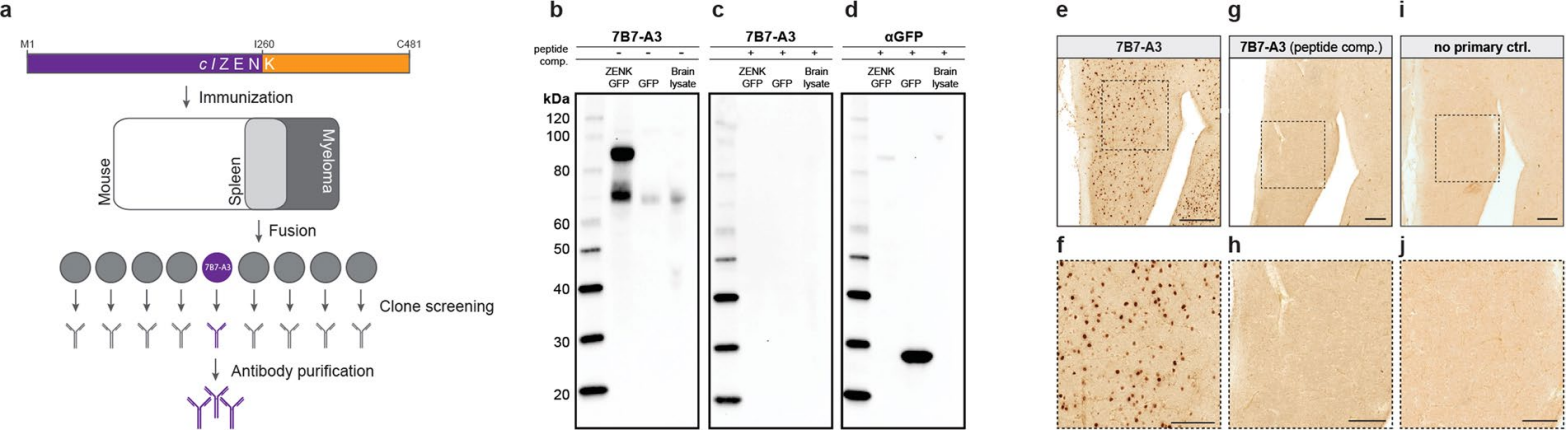
ZENK antibody generation and validation. (**a**) Diagram depicting the methodology employed to generate the ZENK antibody. A 260 amino acid N-terminal fragment (purple) of the pigeon ZENK protein (orange) was recombinantly expressed and injected into mice. Harvested spleen cells were fused with myeloma cells and hybridomas screened by western blot analysis. This resulted in the identification of clone 7B7-A3 (shown in purple). (**b**) Western blot analysis of pigeon embryonic fibroblasts expressing ZENK-GFP or GFP alone, and protein lysates from pigeon brain tissue. Blots incubated with 7B7-A3 (left panel) show bands at ~70 kDa and ~95 kDa, corresponding to the endogenous and GFP tagged protein, respectively. (**c**) and (**d**) Peptide competition experiment. Preincubation of the antibody with the antigen (+) prevents binding of 7B7-A3 antibody (**c**), but not of a GFP antibody (**d**). (**e,f**) Permanent immunohistochemical staining using 7B7-A3 leads to labeling of cell nuclei on pigeon brain sections. (**g,h**) Antigen competition experiment. Preincubation of the antibody with the antigen abolishes 7B7-A3 staining. (**i,j**) No primary antibody control. Scalebars represent 100 μm in (**e**), (**g**), and (**i**); and 50 μm in insets (**f**), (**h**), and (**j**).

To determine the full-length protein sequence of ZENK in *Columba livia* (*cl*ZENK) we extracted mRNA from the pigeon brain, generated cDNA, and amplified the transcript using primers designed from existing genomic resources^31,32^. This resulted in the cloning of a 1443 bp gene product that codes for a 481 amino acid protein. An N-terminal fragment 260 amino acids in length (1–260) with high surface probability was chosen as the antigen. The protein was heterologously expressed in *E. coli*, purified, the sequence verified by mass spectrometry, and then injected into BALB/c mice. The spleens of immunized mice were harvested, hybridomas generated, and clones screened by western blot analysis for sera that bind the *cl*ZENK protein (Fig. 1a). This resulted in the identification of clone 7B7-A3. Purified antibodies generated from this clone detect ZENK (~70 kDa), and GFP tagged ZENK (~95 kDa) in protein lysates from pigeon embryonic fibroblasts (PEFs) transiently transfected with *cl*ZENK-GFP (Fig. 1b). In lysates generated from the pigeon brain we observed a single 70 kDa band that is consistent with endogenously expressed ZENK. It should be noted that like some commercially available antibodies raised against ZENK the detected size of 70 kDa in western blots exceeds the predicted weight of 57 kDa, possibly due to posttranslational modifications. Therefore to validate the selectivity for the *cl*ZENK epitope, we performed a peptide competition experiment by preincubating the antibody with the antigen. This preincubation prevents binding of the 7B7-A3 antibody (Fig. 1c), but not binding of a GFP antibody control (Fig. 1d). Next, we assessed the utility of the 7B7-A3 antibody as a marker on histological sections. Employing antigen retrieval and a permanent staining protocol we found that 7B7-A3 antibody reliably labels cell nuclei in pigeon brain sections at dilutions as low as 1:500,000 (3.2 ng/ml), without the need for post-chromogenic enhancement (Fig. 1e,f). Pre-absorption of the 7B7-A3 antibody with the antigen (Fig. 1g,h), as well as omission of the primary antibody (Fig. 1i,j) resulted in no visible nuclear staining, confirming the selectivity of the 7B7-A3 antibody for the *cl*ZENK epitope. Taken together, these data indicate that the 7B7-A3 antibody binds *cl*ZENK.

### Distribution of ZENK in the pigeon brain

**Figure 2:**
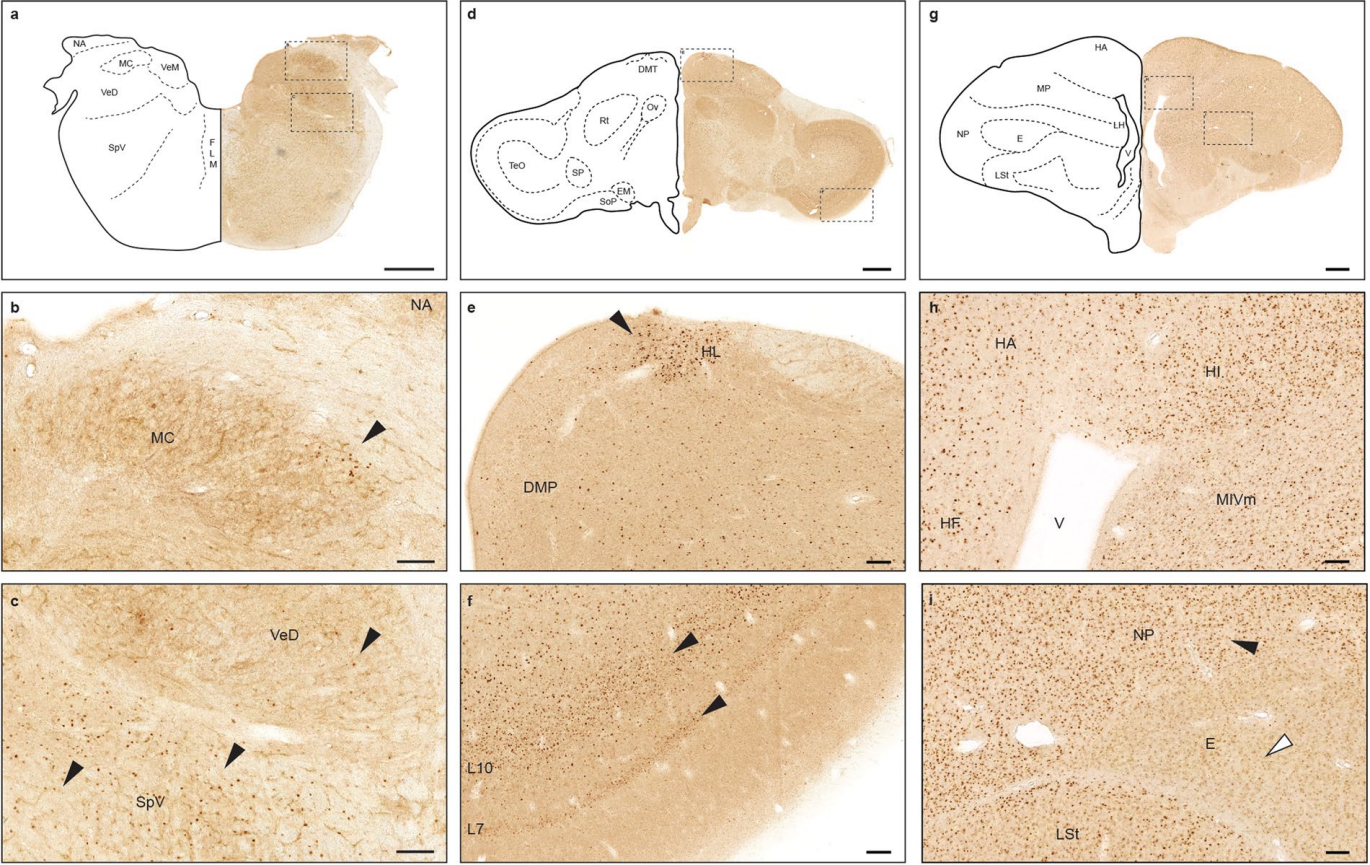
ZENK^+^ neuronal populations in the pigeon brain. Pigeon brain sections at Karten/Hodos coordinates P1.25 (**a**), A5.00 (**d**), and A9.25 (**g**) stained with the 7B7-A3 antibody. Insets (dashed boxes) are shown below the corresponding images. In the absence of stimulation ZENK-positive nuclei can be detected throughout the pigeon brain in the (**b**) nucleus magnocellularis; (**c**) nucleus vestibularis descendens (VeD) and spinal trigeminal nucleus (SpV); (**e**) lateral habenular nucleus (HL) and nucleus dorsomedialis posterior thalami (DMP); (**f**) layer 7 and 10 of the tectum opticum; (**h**) hyperpallium apicale (HA), hippocampal formation (HF), hyperpallium intercalatum (HI), and mesopallium intermedioventrale, pars medialis (MIVm); and (**i**) nidopallium (N) and striatum laterale (LSt). Black arrowheads highlight ZENK positive neuronal populations. The white arrowhead in (**i**) highlights the lack of ZENK staining in the entopallium (E). Scalebars represent 1 mm in upper row and 100 μm in insets.

Next, we examined the distribution of ZENK expressing neuronal populations in the pigeon brain. We sacrificed animals that were maintained in a standard aviary (an environment with numerous sensory cues) and stained sections from the brainstem, thalamic and forebrain regions involved in higher sensory processing using our 7B7-A3 antibody. Within the brainstem we observed ZENK-positive cells in primary nuclei of the auditory (e.g. nc. magnocellularis, Fig. 2a,b), vestibular (e.g. nc. vestibularis dorsalis, Fig. 2a,c), and trigeminal system (e.g. SpV, Fig. 2a,c). In the midbrain, the 7B7-A3 antibody labels thalamic relays, such as the lateral habenular nucleus (Fig. 2d,e) as well both retinorecipient and nonretinorecipient layers of the rostral optic tectum (Fig. 2d,f). In the forebrain, consistent with previous studies^21–23^, we observed limited immunoreactivity within entopallial structures (Fig. 2g,i). In contrast, we found robust and broad ZENK staining in other areas involved in higher sensory processing such as the rostral piriform cortex^33^ and multi-sensory integrating regions of the medial intermedioventral mesopallium^34^, as well as the apical hyperpallium^35^ and the rostral hippocampal formation^36^ (Fig. 2g,h). This broad ZENK expression was markedly reduced after isolating the animals (n = 3) for 7 hours in a dark cage in silence. ZENK^+^ cells were reduced by 72% in the rostral hippocampus (P < 0.05; t = 2.45; df = 4; Supplementary Fig. 1a–c), by 82% in the lateral striatum (P < 0.05; t = 3.47; df = 4; Supplementary Fig. 1d–f), and by 71% in the optic tectum (P < 0.05; t = 3.70; df = 4; Supplementary Fig. 1g–i). We conclude that ZENK is expressed in various networks of the pigeon brain, including neuronal populations of the vestibular, trigeminal, and auditory, and visual system, thalamic relay stations, and telencephalic structures. This provides an important entry point for the mapping of functional activity of these networks using 7B7-A3.

### The 7B7-A3 antibody is a neuronal activity marker

**Figure 3:**
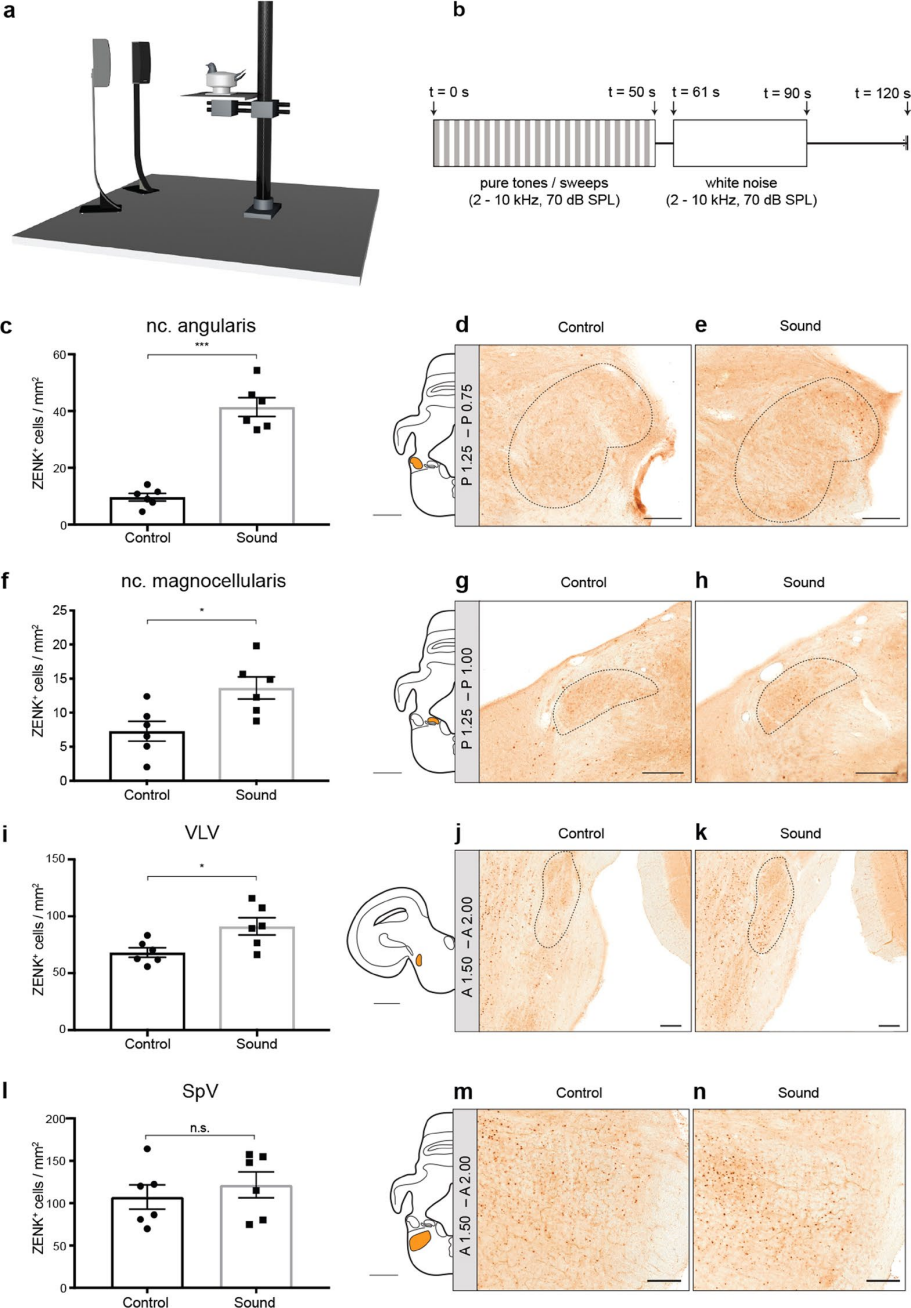
The 7B7-A3 antibody is a neuronal activity marker. (**a**) Set-up for auditory activation experiment. Pigeons were immobilized in a body harness and exposed to high frequency sounds delivered through two speakers at 1.5 m distance to the animals (n = 6) or to no sound (n = 6, Control). (**b**) Sound stimulation paradigm: T1-t50: double-pulsed pure tones and sweeps (each 50 ms, 2–10 kHz, 70 dB SPL), delivered in a pseudorandomized order; t51-t60: silence; t61-t90: white noise (2–10 kHz, 70 dB SPL); t91-t120: silence. The sound file was looped 30 times to achieve a 60 min stimulation. (**c**–**e**) Quantification of ZENK^+^ cells per mm^2^ in the nucleus angularis, (**f**–**h**) nucleus magnocellularis, (**i**–**k**) nucleus ventralis lemnisci lateralis (VLV), and (**l**–**n**) spinal trigeminal nucleus (SpV). Right panels show representative coronal sections from control and stimulated animals stained with the 7B7-A3 antibody as well as the segmented regions for the nucleus angularis (**d,e**), nucleus magnocellularis (**g,h**), VLV (**j,k**), and SpV (**m,n**). On the left of (**d**), (**g**), (**j**), and (**m**), anatomical drawings of coronal hemisections with the segmented nuclei are shown in orange and stereotaxic coordinates according to Hodos and Karten^50^ are indicated in the grey boxes. Data are presented as mean +/− SD. *P < 0.05; ***P < 0.0001, one tailed t-test after Bonferroni-correction in auditory areas. n.s.: not significant. Scalebars represent  2 mm in the drawings and 200 μm in stained sections.

To assess whether the 7B7-A3 antibody can be used to detect stimulus-induced ZENK expression, we challenged restrained pigeons (n = 6) with a 60 min auditory stimulus consisting of pure tones, sweeps, and white noise (2–10 kHz, Fig. 3a,b). Control animals were kept for 60 min in silence (control, n = 6). The birds were then immediately perfused, the brains sliced, and matched sections containing the cochlear nuclei (i.e. the nuclei angularis, NA, and magnocellularis, NM) were stained with the 7B7-A3 antibody. As the NA and NM represent the first stations of the central auditory pathway in the pigeon and receive direct input from cochlear afferents, we predicted that sound exposure would lead to an increase in ZENK positive cells in these brain regions. Blind quantitation revealed a 4-fold increase in ZENK positive cells in the NA when comparing stimulated and control animals (P < 0.0001; t = 8.964; df = 10; Fig. 3c–e). Consistent with the proposed tonotopic organization within the NA in birds^37^, the high frequency sound-induced activation was most pronounced in the anterolateral divisions of the nucleus. Similarly, we found a significant upregulation of ZENK positive cells in the rostral parts of the NM, although ZENK expression was sparse in this area (P < 0.05; t = 2.891; df = 10; Fig. 3f–h). As the NA and NM project directly to the lemniscal complex in the caudal mesencephalon, which is the first station receiving binaural information, we also quantified ZENK expression in the nucleus ventralis lemnisci lateralis (VLV). Again, we found a significant increase in ZENK positive cells in stimulated animals (P < 0.05; t = 2.668; df = 10; Fig. 3i–k). We did not observe an increase in ZENK positive cells in the non-auditory trigeminal nucleus SpV in response to sound (P > 0.05; t = 0.690; df = 10; Fig. 3l–n). Together, these results show that the 7B7-A3 antibody can be employed as a marker for stimulus-induced neuronal activity.

### 7B7-A3 labeling in other birds

**Figure 4:**
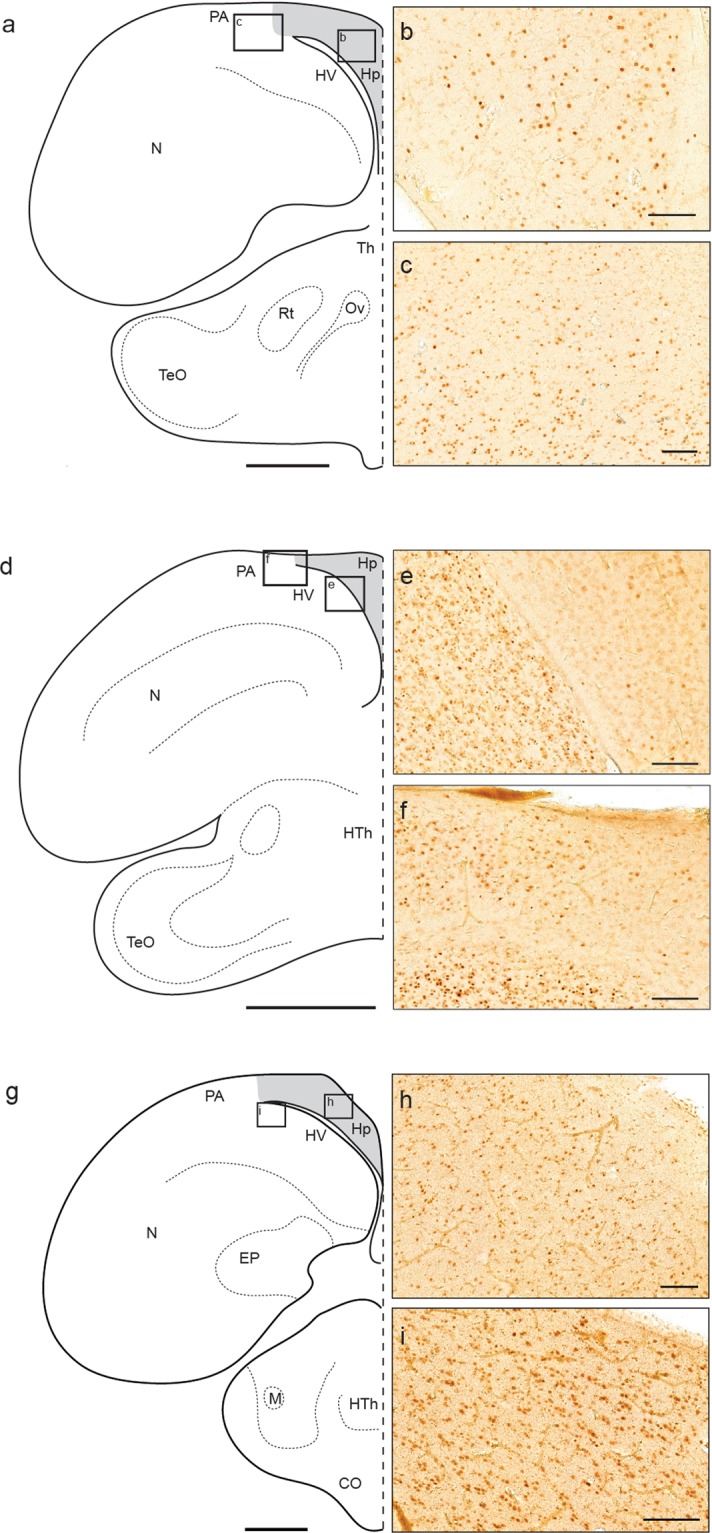
Hippocampal 7B7-A3 labeling in different avian species. Schematic representations of a coronal brain section of the (**a**) rock pigeon, (**d**) zebra finch, and (**g**) domestic chicken. The hippocampus is highlighted in grey; insets are shown on the right. Staining with 7B7-A3 antibody in the pigeon leads to robust nuclear staining within the entire (**b**) hippocampus and (**c**) parahippocampal area. In the zebra finch, hippocampal neurons show only sparse ZENK staining (**e**), while robust nuclear signal can be detected in the mesopallium as well as in the (**f**) parahippocampal area. In the chicken, 7B7-A3 stains the (**h**) hippocampus uniformly, but less intensely than those cells in the (**i**) parahippocampal area. Scalebars represent 2 mm in (**a**), (**d**), and (**g**); and 100 μm in (**b**), (**c**), (**e**), (**f**), (**h**), and (**i**).

Having established 7B7-A3 as a reliable marker for neuronal activity in the pigeon, we tested whether the antibody is compatible with staining in other avian model species. Using our standard immunohistochemical labeling procedure, we assessed the staining quality in the pigeon, zebra finch (*Taeniopygia guttata*), and domestic chicken (*Gallus gallus domesticus*). We collected brain sections from all birds (n = 3) and focused on the distribution of ZENK positive cells in the caudal hippocampus and surrounding structures. In pigeons we observed ZENK positive nuclei in all divisions of the caudal hippocampus as well as in the parahippocampal area (Fig. 4b,c). Using 7B7-A3 at the same concentration in the zebra finch (1:500,000) we again observed nuclear staining. Compared to pigeons this staining was sparser in the hippocampus, but more pronounced in the ventrally located mesopallium (Fig. 4e) and the parahippocampal area (Fig. 4e,f). In the chicken, a higher concentration of the antibody (1:5000) was required to achieve immunolabelling in the hippocampus (Fig. 4h). There, ZENK positive cells were less intensely stained, but again showed nuclear signal. Similarly to the zebra finch, the expression in mesopallial regions was more pronounced (Fig. 4i). While context dependent ZENK expression could account for the observed differences in ZENK distribution between the species, these results confirm that 7B7-A3 is compatible with immunohistochemical detection of ZENK in the rock pigeon, zebra finch, and chicken, thereby broadening the utility of the 7B7-A3 antibody.

## Discussion

Here, we present a new monoclonal antibody (7B7-A3) that targets the ZENK protein from the rock pigeon. This antibody has several salient features. First, it is the only antibody that has been specifically raised against a bird ZENK protein. The antibody is compatible with western blot analysis and immunohistochemistry and shows a robust signal to noise ratio under denaturating conditions. Importantly, selective binding of the antibody to the antigen (as recommended by the international working group for antibody validation^38^) demonstrates that it binds to ZENK. Second, the 7B7-A3 antibody exhibits high sensitivity for its target, as evident from the immunohistochemical results. On pigeon brain tissue sections, a dilution of 1:500,000 is sufficient to label neuronal nuclei without intensification by heavy metals. This high sensitivity ensures thorough and quantifiable labeling of activated brain areas, but also the identification of neuronal populations in which ZENK expression has not been described before, such as the nuclei angularis and magnocellularis. Third, the wide distribution of ZENK positive cells in the pigeon brainstem detected by the 7B7-A3 antibody make it an ideal tool to study the functional organization of many sensory entry stations, such as the trigeminal, auditory, or vestibular systems. For instance, the anatomical localization of sound-induced activity in the pigeon has so far been focused on telencephalic structures such as Field L^39^ or the nucleus basalis^40^ and in the midbrain on the nucleus mesencephalic lateralis pars dorsalis^41^. Challenging the assertions that IEG induction does not occur in the cochlear nuclei of birds^12^, we have shown the 7B7-A3 antibody does label the pigeon auditory brainstem in an activity-based manner. Fourth, 7B7-A3 recognizes ZENK epitopes from different bird species, broadening its utility to numerous research fields associated with the avian brain. Studies of the zebra finch song system have greatly benefitted from ZENK mapping^8,12^, and in the pigeon, ZENK induced by homing behavior has been used to study the neuronal correlates of navigational processes^29,42^. Finally, with the advent of brain clearing methods, such as CUBIC^43^, CLARITY^44^, or iDISCO^45^, the 7B7-A3 antibody may also serve as a useful tool to investigate IEG expression on a global scale^46,47^. Such experiments will provide further insight into the neuronal circuits that mediate the complex repertoire of avian behaviours. Previous studies that have employed ZENK as a surrogate marker for neuronal activity have primarily relied on a polyclonal antibody (C-19), which has now been discontinued by the supplier^27,28^. We therefore anticipate that the 7B7-A3 ZENK monoclonal antibody described in this manuscript will be a valuable tool for the avian neuroscience community. This antibody is available on request.

## Methods

### Identification and amplification of the *cl*ZENK sequence

Brain tissue samples were collected from adult rock pigeons (*Columba livia*), snap frozen in liquid nitrogen, and mechanically homogenized in a Tissue Lyser (Qiagen, 85300). Following the manufacturers’ instructions, total RNA was extracted from the lysates using the RNeasy mini kit (Qiagen, 74104) and reversely transcribed to cDNA using the Superscript III cDNA synthesis SuperMix kit (Invitrogen, 18080400). The resulting pigeon brain cDNA library was diluted to a working concentration of 1:100 and stored at -20 °C. Sets of overlapping polymerase chain reaction (PCR) primers for the predicted *cl*ZENK transcripts were designed based on available genomic resources. PCR amplification of the pigeon brain cDNA library was performed in two segments with the Phusion Hot Start Flex DNA polymerase (NEB, M0535S) using the following primer sequences: segment 1: Fwd: ATGGACACTCACTATCCCAA, Rev: GCAAACGCCTTAATAGTGG; segment 2 Fwd: CCACTATTAAGGCGTTTGC, Rev: GGACAACTGAGATTTGCTGA. PCR products were evaluated by agarose gel electrophoresis, TA-cloned into the pCR4-TOPO vector (Invitrogen, K457502) following the manufacturer’s protocol, and analyzed by Sanger sequencing. For protein expression, a C-terminal enhanced green fluorescence protein (eGFP) tag was fused to the total *cl*ZENK nucleotide sequence after a four residue glycine-serine linker and cloned into the mammalian expression vector pCIneo. Gibson assembly was performed according to the manufacturer’s (MBS VBC) protocol with the following fragments: pCIneo plasmid linearized with NheI/XhoI restriction enzymes (NEB, R0131S and R0146S); *cl*ZENK amplified in two fragments using Phusion Hot Start Flex DNA polymerase; eGFP amplified from a host plasmid using Phusion Hot Start Flex DNA polymerase. The full coding sequence of the construct was verified by Sanger sequencing.

### Monoclonal antibody generation

For the generation of a monoclonal antibody against *cl*ZENK, a 260 amino acid protein fragment (1–260) was selected as antigen; the codon-optimized mRNA was synthesized and cloned into the pET23a(+) expression vector including a C-terminal hexahistidine tag by an external contractor (GenScript USA Inc). The peptide was recombinantly expressed in Lemo21(DE3) bacteria (NEB, C2528J) and purified from the insoluble fraction of the bacterial lysates in 8 M urea over a Nickel-sepharose column (GE Healthcare, 17526801) and the Whatmam^TM^ Elutrap^TM^ electroelution system (Thermo Fischer, 15560753). The eluate was verified by mass spectrometry.

BALB/c mice were immunized with the purified antigen. Following the hybridoma technique^48^, monoclonal antibody producing cells were generated and positive clones were isolated after several rounds of monoclonal selection by testing cell culture supernatants for immune reactivity in western blots and on tissue sections (see below). Hybridoma cells of the final clone, 7B7-A3, were weaned from HAT (Gibco, 21060017) in a stepwise process over 2 weeks. For antibody expression, a hollow fiber cartridge (Fiber Cell Systems, C2011) was inoculated according to manufacturer’s instructions and the cells were cultivated using production medium containing DMEM (Media kitchen, VBCF) supplemented with 5% FCS (Gibco, 16140071), 1% Penicillin-Streptomycin (Gibco, 15140122), 2 mM L-Glutamine (Gibco, 25030081), and 1 mM sodium pyruvate (Gibco, 11360070). Supernatants were harvested and pooled for 5 weeks; the monoclonal antibody was isolated by affinity purification using a Protein G column (elution buffer: 100 mM Glycine, 100 mM NaCl, pH 2.46).

### Cell culture, protein expression and western blot analysis

Primary cell cultures of pigeon embryonic fibroblasts (PEF) isolated at embryonic day 10 were cultured at 37 °C and 5% CO_2_ on 10 cm cell culture dishes (Eppendorf) in DMEM (MBS VBC) supplemented with 8% chicken serum (C5405), 2% FCS (Gibco, 16140071), 1% penicillin-streptomycin (Gibco, 15140122), 1% L-Glutamine (Gibco, 25030081), 1% non-essential amino acids (Gibco, 11140050), sodium pyruvate (Gibco, 11360070), and 70 mM β-mercaptoethanol (Merck, 805740). Cells were transfected at ~80% confluence with 20 μg pCIneo-ZENK-eGFP or a control construct (pCIneo-GFP) using Lipofectamine 2000 transfection reagent (Invitrogen, 11668019) according to the manufacturer’s instructions. 24–48 h post transfection, cells were washed twice in PBS and harvested in 250 μl RIPA buffer containing 150 mM NaCl, 1% Triton X-100 (Sigma Aldrich, X100), 0.5% sodium deoxycholate (Sigma Aldrich, D6750), 0.1% SDS (Sigma Aldrich, 862010), 50 mM Tris pH 8, and one protease inhibitor tablet (Pierce, A2963). Cells were snap frozen in liquid nitrogen, thawed, lysed for one hour shaking at 4 °C, and centrifuged for 30 min at 4 °C (16200 g). Supernatants were collected and total protein concentrations were determined using the BCA Protein Assay kit (Pierce, 23225). For western blot analysis, 20 μg of protein extracts were denatured in Laemmli sample buffer (BIO-RAD, 1610747) for 5 min at 95 °C and separated in a 4–12% bis-tris poly-acrylamide gel (Invitrogen. NP0322). Gel electrophoresis was performed for 2 h at 120 V in MOPS SDS running buffer (Invitrogen, NP0001) and subsequent transferred to a PVDF membrane (Merck, ISEQ. 00010) for 3 h at 100 V and 4 °C in transfer buffer (30 mM tris, 240 mM glycine, 0.025% SDS). After rinsing in Tris-buffered saline supplemented with 0.1% Tween-20 (Sigma Aldrich, P1379) (TBST) and blocking for 1 h in TBST supplemented with 5% non-fat milk powder, membranes were incubated in primary antibody solution composed of 5% milk/TBST over night at 4 °C. The following concentrations were used: 1:500 (mouse serum screen), 1:2000 (7B7-A3 supernatant), 1:500 GFP (2B6, Monoclonal Antibody Facility, Max Perutz Laboratories). After washes in TBST, membranes were incubated for 1 h with the respective HRP-tagged secondary antibody (Abcam, ab6823) in 5% milk/TBST, washed, and imaged using the ECL western blotting detection reagents (Amersham, RPN2232) on a BIORAD ChemiDOC^TM^ imager. For the antigen competition experiment, 4 μg of the final ZENK antibody was incubated with 20 μg of the immunogen for 2 h, before the antibody-antigen solution was used as described above in primary antibody solution.

### Avian tissue collection for immunohistochemistry

Adult pigeons (*Columba livia*) were maintained on a 12:12 light-dark cycle at 25 °C in a custom-built aviary. Zebra finches (*Taeniopygia guttata*) and domestic chicken (*Gallus gallus domesticus*) were purchased from local suppliers and kept in individual transport boxes for at least 1 hour prior to sacrifice. Animals used to assess ZENK expression in the absence of sensory stimulation were individually housed for at least 7 hours in total darkness and silence. For brain tissue collection birds were sacrificed and transcardially perfused with 200 ml sterile filtered, warm PBS supplemented with 15 kU Heparin (Carl Roth, 7692.2) followed by 200 ml of 4% PFA in phosphate buffer (Sigma Aldrich, 158127). The brains were dissected, postfixed in 4% PFA for 18 hours at 4 °C, dehydrated in 30% sucrose in 0.1 M PBS (Sigma Aldrich, S9378) for 3 days at 4 °C, and mounted on a sledge microtome. 40 μm serial sections were acquired in the coronal plane and stored in anti-freeze composed of PBS supplemented with 30% Glycerol (AppliChem, A0970) and 30% Ethyleneglycol (Sigma Aldrich, 324558) at −20 °C. For broad assessment of basal ZENK expression in the pigeon, at least 16 tissue sections were mounted on glass slides (Thermo Fischer Scientific, J4800AMNZ). For the comparison of ZENK expression in the pigeon, zebra finch, and chicken, individual hippocampal sections of each species were mounted on the same slide. For all experiments at least three animals were used. All sections were dried over night before proceeding with immunohistochemical staining.

### Ethical framework

All experiments were carried out in accordance with relevant guidelines and regulations. Experimental protocols were approved by an existing ethical framework (GZ: 214635/2015/20) granted by the City of Vienna (Magistratsabteilung 58).

### Auditory activation

Sound activation experiments were performed in an experimental chamber in darkness. Birds were habituated to a customised body harness and experimental room for 30 minutes on 3 consecutive days prior to the experiment. The night prior to the experiment birds were singularly housed. Single animals were placed in the center of the chamber in the body harness on an elevated platform, with 2 speakers (Sony, SS-MD333) placed 1.5 m away from the birds. Two birds were tested on each day and were presented with either a 60 min sound file or no sound. The experimenter was blind to the stimulation condition at all times. The sound stimulus was generated using the open source software Audacity (D. Mazzoni) and consisted of a 2 min high activity sequence that was looped 30 times to achieve a 60 min stimulation^49^. The sequence was composed of: (*start to t* = *50 s*): double-pulsed sine waves (each 50 ms, 2–10 kHz, 70 dB SPL) and sweeps (each 50 ms, 2–10 kHz, 70 dB SPL) delivered in a pseudorandomized order; (*t* = *51 s to t* = *60 s*): silence; (*t* = *61 s to t* = *90 s*): white noise (2–10 kHz, 70 dB SPL); (*t* = *91 s to t* = *120 s*): silence. A habituation period prior to stimulation to exclude handling-induced ZENK expression was omitted due to the strong activation profile of the sound stimulus. Following stimulus presentation birds were immediately sacrificed and perfused and brains were processed as described above. For the analysis of sound-induced ZENK expression in the auditory brainstem, every third section of the cochlear nuclei was mounted on glass slides starting the at the caudal border of the nucleus magnocellularis^50^ (P 1.50). In addition, five sections of the isthmic brainstem containing VLV were mounted starting at A 1.50. All sections were dried overnight before proceeding with immunohistochemical staining.

### Immunohistochemistry

Mounted and dried sections were washed 3 × 5 min in PBS before heat-based antigen retrieval was performed to unmask fixed antigens following the manufacturer’s instructions (Vector Laboratories, H-3301). After a 30 min cooling period, slides were washed 3 × 5 min in PBS and incubated in primary antibody diluted in 0.3% Triton-X100/PBS with 4% milk powder over night at room temperature. The following concentrations were used: 1:5 (mix clone supernatants), 1:100 (final clone supernatant), 1:500,000 (7B7-A3, pigeon), 1:500,000 (7B7-A3, zebra finch), 1:5000 (7B7-A3, chicken). The next day, slides were washed 3 × 5 minutes in PBS, incubated with the secondary antibody (1:1000, anti-mouse, Vector Laboratories, PK-6100) for 2 hours at room temperature, washed, and incubated with the Vectastain Elite ABC HRP reagent (Vector Laboratories, PK-6100) for 1 h. After another round of washing, slides were incubated for 1 min in PBS supplemented with 0.06% Diaminobenzidine (Sigma Aldrich, D5905) and 0.08% H_2_O_2_. After a final washing step, slides were dehydrated in serial dilutions of ethanol and cover slipped.

### Counting and statistical analysis

Slides were scanned on a slide scanner with a 20x objective (Pannoramic 250 Flash III, 3DHistech). All auditory nuclei were identified based on surrounding white matter tracts and segmented manually (at least 8 bilateral segments from 4 sections) using Pannoramic Viewer (Pannoramic Viewer 1.15.4, 3DHistech). ZENK expression in SpV was assessed on 3 sections (6 bilateral segments). Automated identification and counting of ZENK positive nuclei on exported segments were performed by custom made rule sets using a machine-learning algorithm embedded in the Definiens Architect software (Definiens Architect XD, Definiens Software). The number of ZENK positive cells per mm^2^ was calculated based on the area of exported segments. Context dependent changes in ZENK expression were assessed using a one-sided t-test and sound-induced changes in ZENK expression were assessed using one-sided t-tests corrected for multiple comparisons (Bonferroni) in auditory areas. Statistical analyses were performed in R (Team 2013) and figures were generated using Graphpad Prism 8 (Graphpad Software Inc).

## Supplementary information

Supplementary Information.

## Data Availability

The datasets generated during and/or analysed during the current study are available from the corresponding author on reasonable request.
