# Supplementary material for: A high sensitivity ZENK monoclonal antibody to map neuronal activity in Aves
Source: Sci Rep. 2020 Jan 22;10:915. doi: 10.1038/s41598-020-57757-6 (PMC6976653; doi:10.1038/s41598-020-57757-6)
Supplement: Supplementary file 1 — Supplementary Information. [file 41598_2020_57757_MOESM1_ESM.pdf]

Supplementary Information

A high sensitivity ZENK monoclonal antibody to map neuronal activity in *Aves*

Gregory Charles Nordmann, Erich Pascal Malkemper, Lukas Landler, Lyubov Ushakova, Simon Nimpf, Robert Heinen, Stefan Schuechner, Egon Ogris, and David Anthony Keays

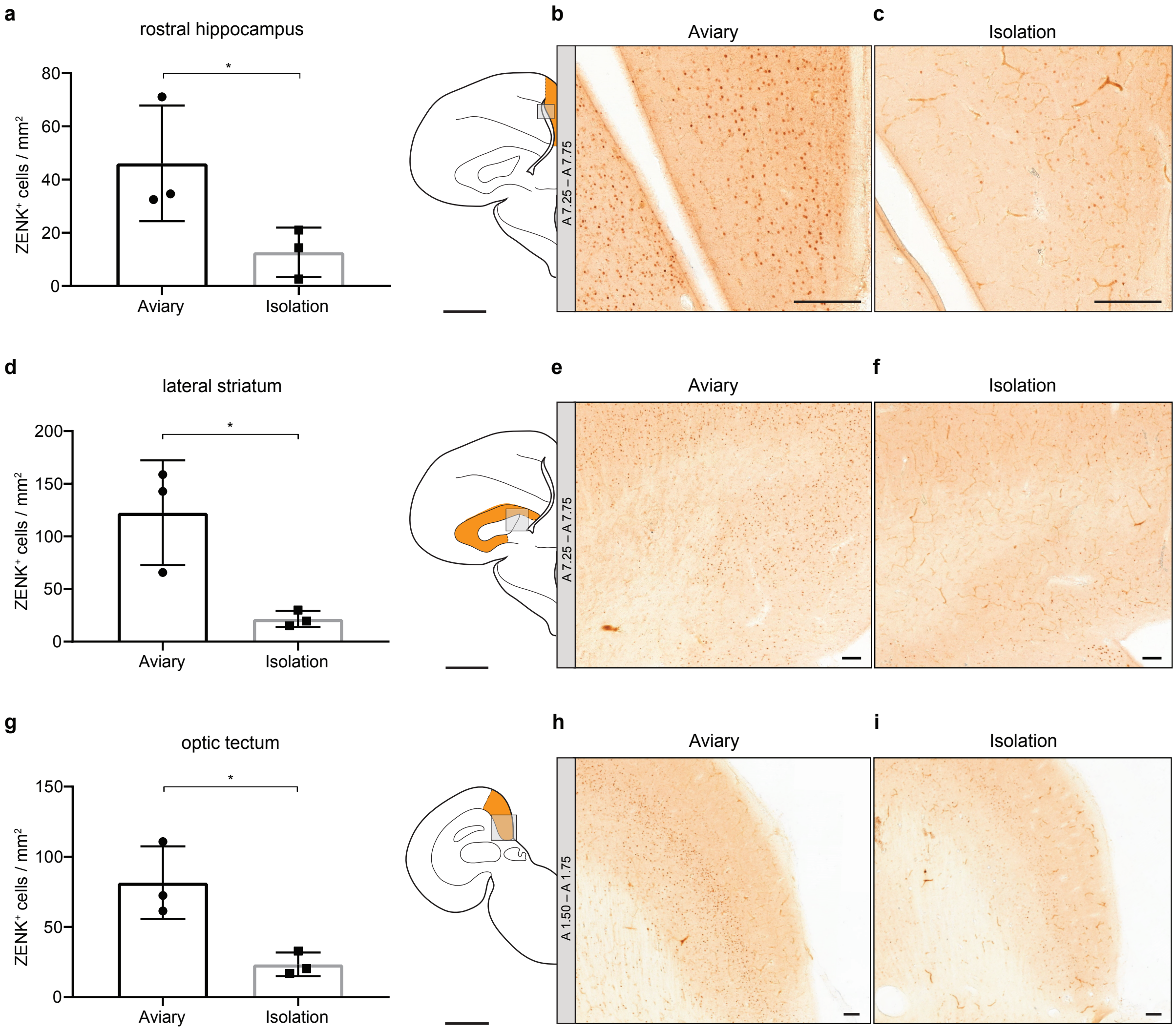

Supplementary Figure 1. Effect of sensory deprivation on ZENK expression. Birds were kept under standard conditions (aviary; n = 3) or in a dark cage in silence (isolation; n = 3) for at least 7 hours. (**a-c**) Quantification of ZENK$^+$ cells per mm^2 in the rostral hippocampus, (**d-f**) lateral striatum, and (**g-i**) optic tectum. In isolation ZENK$^+$ cells were reduced by 72% in the rostral hippocampus ($p < 0.05$; $t = 2.45$; $df = 4$), by 82% in the lateral striatum ($p < 0.05$; $t = 3.47$; $df = 4$), and by 71% in the optic tectum ($p < 0.05$; $t = 3.70$; $df = 4$). Right panels show representative coronal sections for both conditions stained with the 7B7-A3 antibody. Respective anatomical drawings of coronal hemisections with the segmented areas are shown in orange and stereotaxic coordinates are indicated in the grey boxes. Data are presented as mean +/- SD. *$p < 0.05$, one tailed t-test after Bonferroni-correction. Scalebars represent 2 mm in the drawings and 100 µm in stained sections.
